# Racialized ethnic boundary-making in multilingual Kazakhstan: from silent to recognitional tolerance

**DOI:** 10.3389/fsoc.2026.1890041

**Published:** 2026-09-03

**Authors:** Assem Tastanova, Olzhas Karibayev, Yerkebulan Yerzhanov, Akerke Raiymbekova, Galym Malik

**Affiliations:** 1 Department of Social Sciences, Abai Kazakh National Pedagogical University, Almaty, Kazakhstan; 2 Department of International Relations, Kazakh Ablai Khan University of International Relations and World Languages, Almaty, Kazakhstan; 3 Department of Social Sciences, Asfendiyarov Kazakh National Medical University, Almaty, Kazakhstan

**Keywords:** civic identity, ethnic boundaries, Kazakhstan, linguistic capital, multilingualism, race and ethnicity, racialization, recognition

## Abstract

This conceptual analysis examines interethnic tolerance in Kazakhstan as racialized ethnic boundary-making in multilingual and civic life. Drawing on an author-led thematic-conceptual synthesis of published scholarship and open legal, statistical, and institutional sources, it does not present new primary data or impose a Western biological definition of race. Racialization is used relationally: ethnic, linguistic, regional, migratory, visible, and ethnocultural signs become racialized when treated as durable indicators of competence, loyalty, modernity, or belonging. The article develops a four-mechanism model—linguistic accommodation, civic framing, institutional recognition, and normalization through interaction—and distinguishes silent tolerance from recognitional tolerance. Silent tolerance denotes polite coexistence, conflict avoidance, and official harmony that leave unequal status unspoken; recognitional tolerance requires difference to be acknowledged as equally legitimate in law, institutions, and everyday interaction. The source-based synthesis suggests that choices among Kazakh, Russian, English, and minority languages may soften boundaries but may also reproduce hierarchy through accent evaluation, qandas positioning, minority visibility, and unequal access to mobility languages. Contrasts with Quebec, Belgium, and the Baltic states clarify how multilingual stability can coexist with unequal participation. The article contributes by showing how racialized boundaries can be theorized where race is not a dominant public category and by providing indicators and testable propositions for future empirical research.

## 1 Introduction

Kazakhstan is a multiethnic and multilingual post-Soviet society and an important but still underused case for international race and ethnicity research. Ethnic relations are shaped by the state status of Kazakh, the official and everyday use of Russian, the growing role of English in education and professional mobility, the discourse of a civic “Kazakhstani” identity, and institutions that formalize interethnic harmony. These features make Kazakhstan especially useful for studying how social peace is produced in practice. They also show why tolerance cannot be understood only as “friendship of peoples,” the absence of open conflict, or official rhetoric of harmony. Such formulas describe a visible political outcome, but they do not explain the everyday work through which boundaries are softened, displaced, silenced, or reproduced.

For a Race and Ethnicity readership, the case requires careful conceptual translation. Kazakhstan is not organized around race in the same way as settler-colonial, Atlantic, or post-imperial Western societies. Public categories more often operate through ethnicity, language, region, genealogy, citizenship, religion, and post-Soviet nationality classifications. The article therefore does not claim that Kazakhstan has a racial order identical to that of the United States, Britain, France, or South Africa. Rather, it uses racialization in a relational and sociological sense, following Murji and Solomos (2005), Omi and Winant (2015), and Goldberg (2002): selected signs of difference become racialized when they are treated as socially consequential markers of belonging, worth, competence, modernity, loyalty, or threat. In Kazakhstan, such signs may include ethnic origin, language repertoire, accent, visible Central Asian or Slavic appearance, “Russian-speaking” or “Kazakh-speaking” status, rural or urban speech styles, qandas background, and membership in minority communities such as Uyghur, Uzbek, Dungan, Korean, German, Tatar, and others.

This clarification matters because a manuscript on Kazakhstan may otherwise appear to be only about language policy or civic identity. The core argument is different. Language choice and civic identity are analyzed here as mechanisms through which racialized ethnic boundaries are made, negotiated, and normalized. A person's language repertoire may function as communicative ability, but it can also become an index of class position, national loyalty, urban culture, educational capital, or minority status. Likewise, civic identity may create a shared framework of belonging, but it may also conceal unequal access to recognition if minority difference becomes visible mainly in celebratory, folkloric, or state-managed forms.

Existing studies have examined Kazakhstan's ethnic policy, language politics, and nation-building (Dave, 2007; Fierman, 1998, 2006; Smagulova, 2006, 2008). The absence of large-scale ethnic conflict has often been explained through elite framing, official harmony rhetoric, and managed institutional pluralism (Schatz, 2000; Oka, 2007; Laruelle, 2021; Adams, 2010; Cummings, 2012). Work on language and youth identity shows that multilingual repertoires, civic identity, and ethnolinguistic self-understandings are changing (Rees and Webb Williams, 2017; Tlepbergen et al., 2025; Zharkynbekova et al., 2025a,b). Studies of national belonging and immigrant group perceptions further suggest that language-based and ethnocultural identities shape perceived threat, benefit, and attachment (Sharipova, 2020; Samekin et al., 2025). Repatriation studies show that qandas Kazakhs occupy an ambivalent position as both co-ethnics and outsiders requiring integration (Diener, 2009; Bonnenfant, 2012). These literatures remain partly separated. This article brings them together by treating everyday tolerance as boundary work at the intersection of language, civic belonging, institutional recognition, and unequal status.

Kazakhstan's position in the post-Soviet comparative space makes this question analytically significant. Ukraine, the Baltic states, Kyrgyzstan, Azerbaijan, and other post-Soviet societies show that language and ethnicity can be politicized through different pathways. Kazakhstan seeks to manage difference without turning it into open conflict, relying on multilingual repertoires, state-led harmony institutions, and civic framing. It should therefore be analyzed neither as a simple model of harmony nor as a space of hidden domination, but as a social order moving between silent and recognitional tolerance.

The article starts from three research gaps. First, although Kazakhstan's official institutions of interethnic harmony and elite discourse have been described, less attention has been paid to how racialized ethnic boundaries are softened or reaffirmed at the everyday micro-level. Second, although language policy and multilingualism have been studied extensively, concrete choices among Kazakh, Russian, English, and minority languages have not been systematically explained as mechanisms of boundary-making and minoritization. Third, while “Kazakhstani” civic identity has been analyzed, its relationship to everyday tolerance remains theoretically underdeveloped: does civic identity recognize ethnic difference, translate it into a shared political category, or sometimes make inequality less visible?

The aim of the article is to propose a conceptual model that explains everyday tolerance in Kazakhstan's multilingual society as racialized ethnic boundary work. Everyday tolerance does not simply mean that people avoid confrontation. It refers to a social order produced through language choice, norms of politeness, self-naming, attitudes toward ethnic and linguistic groups, perceptions of institutional equality, digital communication, and the quality of repeated everyday interaction.

The article asks three source-based conceptual questions. First, through which mechanisms does published scholarship indicate that the use of Kazakh, Russian, English, and minority languages can soften, reactivate, or hierarchize ethnic boundaries? Second, how should a “Kazakhstani” civic identity be conceptualized when it widens common belonging yet may depoliticize unequal status? Third, which analytical indicators distinguish interaction that produces equal-status recognition from interaction that sustains polite endurance? These questions concern the explanatory implications of the existing source record, not the prevalence or population-level distribution of the proposed processes. Sections 5.1–5.4 answer them, respectively. Section 7 derives separate testable propositions for future primary research; the present article does not test those propositions.

This article makes three contributions to race and ethnicity scholarship. First, it extends ethnic boundary-making theory by showing how racialized boundaries can operate where race is not a dominant public category. Second, it develops a model linking language choice, civic identity, institutional recognition, and everyday interaction as mechanisms through which tolerance is produced and limited. Third, it distinguishes silent tolerance from recognitional tolerance, moving beyond the simple question of whether Kazakhstan is tolerant or intolerant. The core logic is: language choice becomes socially meaningful through symbolic evaluation; symbolic evaluation activates, softens, or hierarchizes ethnic boundaries; and these boundary processes produce either silent tolerance, based on polite coexistence and conflict avoidance, or recognitional tolerance, based on equal-status recognition.

All illustrative material is derived from cited scholarship and open documents, including studies of language attitudes and Kazakhization (Smagulova, 2006, 2008), Kazakh-medium schooling (Fierman, 1998, 2006), student ethnolinguistic identity (Zharkynbekova et al., 2025a,b), Kazakh–English bilingualism (Tlepbergen et al., 2025), civic and national identity (Rees and Webb Williams, 2017; Sharipova, 2020), immigrant-group perceptions (Samekin et al., 2025), and qandas repatriation (Diener, 2009; Bonnenfant, 2012). None is presented as field observation by the authors. Throughout, “Kazakhstan” denotes the case as represented in those sources; extensions beyond direct evidence are marked as analytical propositions or future tests.

Table 1 summarizes the research gaps and the corrective logic developed in response to them.

**Table 1:** Research gaps and the corrective logic of this article.

Research field	Earlier contribution	Remaining gap	Contribution of this article
Ethnopolitics and nation-building	State discourse and managed harmony have been explained.	Everyday boundary work remains less visible.	Tolerance is analyzed as everyday racialized boundary-making.
Race and ethnicity theory	Boundary-making and racialization explain how categories become consequential.	Kazakhstan is rarely theorized beyond descriptive multiethnicity.	The article applies racialization without imposing Western race categories.
Language and civic identity	Language policy and “Kazakhstani” identity have been studied.	Language choice and civic framing are rarely linked to minoritization.	Language repertoires and civic identity are treated as boundary mechanisms.
Minorities, qandas, and interaction	Minority institutions, repatriation, and contact have been documented.	These strands are rarely integrated into a model of recognition.	Silent and recognitional tolerance are offered as a testable framework.

## 2 Conceptual architecture: boundary-making, racialization, linguistic capital, contact, and recognition

The model uses five concepts, each for a distinct task. Ethnic boundary-making is the anchor: it explains how categories are produced and negotiated rather than taken as fixed group essences. Racialization explains when selected signs of difference become linked to unequal worth, loyalty, modernity, or belonging. Linguistic capital explains why Kazakh, Russian, English, and minority languages carry different symbolic and practical value. Intergroup contact identifies the conditions under which repeated interaction can soften boundaries rather than reproduce them. Recognition theory provides the normative threshold: tolerance becomes stronger when difference is not only endured but treated as equally legitimate. This division of labor keeps the article from becoming a broad inventory of theories and makes each concept serve one step in the model.

Table 2 summarizes the distinct analytical role of each concept in the model.

**Table 2:** Theoretical architecture of the article.

Analytical layer	Main theoretical source	Function in the model	Kazakhstan-related implication
Core theory	Boundary-making theory	Explains ethnicity as boundary-making rather than fixed group essence.	Ethnic difference is activated through language, self-naming, accent, institutions, and interaction.
Racialization/minoritization	Racialization theory	Explains how visible, linguistic, regional, or migratory signs become unequal belonging.	“Russian-speaking,” “Kazakh-speaking,” qandas, migrant, minority, or rural accent may become status markers.
Mediating mechanism	Linguistic capital and indexicality	Shows how linguistic resources become symbolic capital.	Kazakh, Russian, English, and minority languages have different legitimacy, mobility value, and status effects.
Interactional condition	Intergroup contact and social identity theory	Specifies when contact reduces prejudice and when it reproduces inequality.	Mixed environments reduce prejudice only when interaction occurs under equal-status conditions.
Normative endpoint	Recognition and multicultural citizenship theory	Distinguishes mere coexistence from public recognition and status equality.	Harmony is not enough if minority difference remains tolerated but not equally recognized.

### 2.1 Racialization without race: Soviet classification and post-Soviet boundary-making

A central challenge for this article is how to analyze race and ethnicity in Kazakhstan without forcing the case into categories produced by different historical formations. In Kazakhstan, official and everyday classifications more often refer to ethnicity, nationality, language, religion, ancestry, region, and citizenship than to race as a formal public category. The argument therefore does not claim that Kazakhstan has the same racial order as the United States, Britain, France, or South Africa. It asks a more precise sociological question: when do ethnic, linguistic, regional, and migratory signs become socially consequential markers of unequal belonging?

This analytic move is not a metaphorical stretch but a recognized development within racialization scholarship. The concept was formulated precisely to detach the study of unequal belonging from biological notions of race, and it has been extended to religion, language, migration status, and regional origin in settings where the public vocabulary of race is weak or absent (Murji and Solomos, 2005; Goldberg, 2002).

This move is particularly appropriate for the Soviet and post-Soviet context. Hirsch (2002) argues that the Soviet regime possessed concepts of race but did not organize governance through what contemporaries understood as the practice of racial politics; nationality was administered chiefly through ethnographic classification and projects of social transformation. Weitz (2002), from a different position in the same debate, shows how national groups could nevertheless be assigned durable judgments of advancement, loyalty, or threat, while Martin (2001) demonstrates how the “affirmative action empire” institutionalized titular and non-titular categories. Post-1991 Kazakhstan did not simply discard this classificatory inheritance; nation-building reweighted it around a Kazakh titular majority, continuing Russian-language infrastructures, civic “Kazakhstani” discourse, and administratively organized ethnocultural representation (Dave, 2007; Laruelle, 2021). The article therefore treats contemporary language and civic categories as historically sedimented rather than as recent or purely interpersonal distinctions. Its novelty lies in connecting this debated Soviet legacy to present-day boundary-making without equating Kazakhstan with an Atlantic racial order.

Racialization is used here as an analytical process rather than a demographic label. It refers to the attachment of appearance, language, accent, religion, descent, migration status, or cultural practice to judgments about competence, loyalty, modernity, respectability, threat, or civic normality. In this article, such judgments are discussed only when documented in cited scholarship or specified as propositions for future testing; they are not attributed to Kazakhstan's population as a whole.

This use of racialization is distinct from nearby concepts. Ethnicization names the classification of people into ethnic groups. Minoritization describes the positioning of a group as subordinate or marginal. A symbolic boundary marks a social distinction, and a linguistic hierarchy assigns unequal value to different languages or repertoires. Racialized ethnic boundary-making is narrower and more demanding: it occurs when ethnic or linguistic signs are treated as durable, almost natural indicators of worth, loyalty, competence, modernity, or rightful belonging. This distinction explains why the article does not reduce the case to language policy alone.

For empirical purposes, racialized ethnic boundary-making in Kazakhstan should be traced through concrete indicators rather than used as a loose metaphor. These indicators include evaluations of accent and speech register; visible or assumed Central Asian, Slavic, or migrant background; labels such as “Kazakh-speaking,” “Russian-speaking,” “qandas,” “urban,” “rural,” or “not local”; unequal access to Kazakh-, Russian-, English-, or minority-language resources; and institutional situations in which a language repertoire becomes a condition for being treated as competent, loyal, modern, or fully belonging. None of these indicators is racial in a narrow biological sense. They become racialized when they are treated as durable signs of unequal social location.

### 2.2 Ethnic boundary-making: not groups, but boundaries and relations

Barth (1969) proposed understanding ethnic groups not through fixed cultural content, but through social boundaries. This insight is important for Kazakhstan because ethnic categories in everyday life are not always rigid or closed. People participate in several linguistic environments at once, use multiple cultural codes, and foreground ethnic, linguistic, regional, or civic self-identification depending on the situation. Brubaker (1996, 2004) and Brubaker and Cooper (2000) caution against treating nations, ethnic groups, and identities as fixed collective substances; the article therefore analyzes categories as situationally activated boundaries rather than as stable group essences.

Wimmer (2008a,b) develops this logic by describing ethnic boundaries as processes produced at micro-, meso-, and macro-levels. At the macro-level, the state formalizes boundaries through legal language regimes, education policy, migration categories, and civic rhetoric. At the meso-level, schools, universities, media, workplaces, neighborhood institutions, and local communities convert those categories into everyday expectations. At the micro-level, people negotiate boundaries through language switching, responses to jokes, evaluations of accent, use of ethnic labels, silence, avoidance, or ordinary politeness.

The distinction between symbolic and social boundaries proposed by Lamont and Molnár (2002), drawing on Gieryn's (1983) earlier notion of boundary work in the demarcation of legitimate knowledge, is especially important. A symbolic boundary is a category, evaluation, or cultural marker that distinguishes “us” from “others.” A social boundary emerges when that marker becomes attached to material consequences, institutional access, or status. In Kazakhstan, fluency in Kazakh, Russian, English, or a minority language may begin as a communicative difference but become a measure of loyalty, urbanity, professionalism, educational mobility, or full citizenship. The article's central concern is this movement from symbolic difference to social hierarchy.

### 2.3 Linguistic capital and symbolic power

It is impossible to understand ethnic boundaries in a multilingual society without addressing language. Bourdieu (1991) shows that language is not merely a neutral medium of communication but a resource that produces symbolic power and social legitimacy. Language has value within a linguistic market: one language may provide official status, another may facilitate everyday communication, a third may open access to international education and professional mobility, and minority languages may be valued affectively while remaining institutionally weak.

In Kazakhstan, Kazakh represents state and national symbolic capital, Russian represents post-Soviet urban and interregional communicative capital, and English represents access to global education and labor markets. These languages are not interchangeable. Multilingualism therefore does not automatically create equality; it creates different channels of symbolic and practical access. Smagulova (2006, 2008) shows the complex relationship among language policy, Kazakhization, language attitudes, and identity. Strengthening the public status of Kazakh is important for national statehood and historical correction, but in a multilingual environment the same process may be experienced as justice, adaptation, or symbolic pressure. Blommaert's (2010) account of mobile linguistic resources and indexical orders, together with May's (2012) work on language and minority rights, helps frame these repertoires not only as communicative tools but also as politically organized resources of status and access.

The role of English differs from that of Kazakh and Russian. English does not directly mark ethnic belonging, but it can create a new classed boundary through education quality, urban-rural location, digital access, parental capital, and university stratification. Tlepbergen et al. (2025) show that Kazakh-English bilingualism in Kazakhstan is connected to regional variation, technology, daily communication, and language attitudes. This does not empirically prove that English always produces class hierarchy, but it supports the analytical point that English has become a mobility resource whose unequal distribution requires further study. English may temporarily soften the Kazakh/Russian linguistic divide while producing another boundary between those who can access global linguistic capital and those who cannot.

### 2.4 Silent tolerance and recognitional tolerance: the conceptual innovation

The article's main conceptual contribution is the distinction between silent tolerance and recognitional tolerance. It builds on theories of toleration and recognition but names a problem specific to state-managed multilingual diversity: a society can be peaceful, polite, and officially inclusive while leaving unequal status largely unspoken. Walzer (1997) treats toleration as a range of possible attitudes toward difference, from resigned acceptance to stronger forms of endorsement. Forst (2013) understands toleration as a norm structured by objection, acceptance, and rejection. Brown (2006) criticizes tolerance as a discourse that may regulate aversion and mask power. Galeotti (2002) argues that toleration must be linked to the public recognition of difference. These works show that tolerance is not an innocent or self-evidently positive concept.

Silent tolerance refers to an order in which people avoid conflict, maintain politeness, and do not openly articulate ethnic or linguistic inequality. It is not simply negative tolerance, because it is not only a private attitude of non-interference. It is an institutional and interactional order maintained through silence, avoidance, official harmony, and the preference not to discuss difficult difference. Recognitional tolerance, by contrast, refers to a social relation in which difference is not merely endured but publicly acknowledged as equally legitimate in legal, institutional, and everyday settings. Building on Adams (2010) and Cummings (2012), who analyze Central Asian states through culture-as-politics and managed pluralism, this distinction specifies when state-led diversity translates into participation and when it remains symbolic incorporation.

Recognition theory makes this distinction normative. Taylor (1994), Honneth (1995), and Fraser (2000) show that recognition is not only cultural visibility but also status equality and social justice. In this article, recognitional tolerance is therefore not a celebration of diversity alone. It requires equal-status participation, linguistic accessibility, freedom from devaluation, and the possibility of articulating difference without being treated as disloyal or disruptive. The distinction is analytically useful because a society may be stable and peaceful while remaining closer to silent tolerance than to recognitional tolerance. Because this is the article's main conceptual contribution, later sections apply the distinction rather than repeatedly redefining it.

## 3 Logic of the conceptual analysis, source selection, and evidentiary boundaries

This article is a Conceptual Analysis rather than Original Research. It presents no new survey, interview, ethnographic, or digital-corpus data. Its unit of analysis is the published claim or contextual fact, not the resident of Kazakhstan. The article develops a model by comparing theoretical propositions with Kazakhstan-focused scholarship and open legal, statistical, and institutional sources. Statements should therefore be read as interpretive syntheses or testable propositions, not as direct causal findings. The format is appropriate because the contribution lies in conceptual differentiation—especially the distinction between silent and recognitional tolerance—and mechanism specification, while Section 7 identifies how future primary research could test the model (Whetten, 1989).

The source base was assembled through purposive, iterative searches in Google Scholar and, where institutional access permitted, Scopus and Web of Science, followed by backward and forward citation tracking. Search strings combined “Kazakhstan” with “ethnic boundary-making,” “racialization,” “language policy,” “Kazakh Russian multilingualism,” “Kazakhstani identity,” “qandas”/“oralman,” “minority languages,” “interethnic tolerance,” “recognition,” and “multiculturalism.” Comparative searches used “Quebec language policy,” “Belgium territoriality language,” and “Baltic Russian-speaking minorities.” Searches were updated during revision in July 2026. Because the aim was conceptual development rather than exhaustive evidence aggregation, no PRISMA-style screening count is claimed; the final bibliography contains 66 scholarly and open contextual sources.

### 3.1 Selection criteria and mode of analysis

The synthesis followed three steps. First, keyword clusters were aligned with four relationships in the model: the social consequentiality of ethnic categories; the symbolic status of language; the civic framing of belonging; and the institutional and interactional conditions of recognition. Quebec, Belgium, and the Baltic states were selected through a most-different logic because they represent, respectively, protected common-language policy, territorial multilingualism, and post-Soviet state-language integration. The contrasts are used to sharpen mechanisms, not to treat the cases as direct equivalents.

Second, a source was included when it supplied a theoretical mechanism, a reported empirical finding from Kazakhstan or a relevant post-Soviet setting, a legal or statistical contextual fact, or a contrast-case interpretation. Opinion-based and substantively unrelated material was excluded. Foundational pre-2000 works were retained when they established a core concept; where later sources overlapped, priority was given to peer-reviewed, case-proximate, and methodologically explicit studies.

Third, the mode of analysis was author-led thematic-conceptual synthesis rather than formal content analysis or automated coding. The unit of synthesis was the published claim or contextual fact. Because the purpose was conceptual integration rather than frequency estimation, the sources were not subjected to formal frequency-based coding, software-assisted coding, or intercoder-reliability testing. Instead, claims were manually organized by source type, case, data basis, relevant mechanism, and evidentiary status—reported finding, contextual fact, theoretical interpretation, or normative proposition—and compared within and across the four relationships. During revision, generative AI assisted with language editing, structural review, methodological clarification, journal-guideline checking, and selected bibliographic or public-source metadata checks. AI was deliberately excluded from final source selection, interpretive synthesis, and substantive theory-building to preserve the traceability of context-sensitive judgments concerning Soviet and post-Soviet classifications and to keep all analytical decisions attributable to the authors. It did not make final inclusion or exclusion decisions, conduct autonomous thematic coding of the source base, or determine the article's substantive conclusions. Recurring categories—language repertoire, accent evaluation, civic framing, minority institutional visibility, qandas positioning, and quality of interaction—were then aggregated into the four mechanisms presented in Section 5. The procedure seeks conceptual adequacy and traceability rather than exhaustive coverage.

The synthesis relies on three bodies of material. The first consists of theoretical works on ethnic boundaries, racialization, symbolic boundaries, linguistic capital, intergroup contact, multicultural citizenship, tolerance, and recognition. The second consists of academic studies on ethnopolitics, language policy, civic identity, youth ethnolinguistic identity, immigrant group perceptions, qandas repatriation, Russian-speaking identity, and intercultural communication in Kazakhstan. The third consists of open official and contextual sources, including the 2021 national census, the legal language regime, and official demographic estimates.

The Kazakhstan case is treated not as a confirmation of pre-existing theory but as a productive test site. It departs from Atlantic settler-colonial cases in lacking a public vocabulary of race, from Western European immigrant-integration cases in centering an indigenous titular language, and from post-Soviet conflict cases in maintaining sustained interethnic peace. These departures make Kazakhstan analytically valuable because they allow a racialized boundary-making framework to be specified for a society in which race is not a dominant public category and then subjected to primary empirical testing. The present article develops that framework; it does not claim to establish the prevalence or distribution of the proposed processes.

Throughout the article, evidence is labeled by status. Findings from prior studies are attributed to those studies; census, legal, and institutional information is treated as contextual fact; hypothetical examples are identified as analytical diagnostics; and untested extensions are stated as propositions. No composite illustration is presented as an observation by the authors. The model therefore remains grounded in the cited record while marking where primary research is still required.

## 4 Contextual evidence from published and open sources: multiethnicity, language regime, and managed diversity

This section summarizes demographic, legal, and institutional facts reported in cited sources; it does not infer everyday attitudes from those facts. The 2021 national census recorded a population of 19,186,015, including 70.4% ethnic Kazakhs, 15.5% Russians, and 3.2% Uzbeks, alongside Ukrainians, Uyghurs, Germans, Tatars, Azerbaijanis, Koreans, and other groups (Bureau of National Statistics, 2023). These figures establish the coexistence of a Kazakh demographic majority and a wider multiethnic population.

The census also reported that 24.5% of the population knew one language, 44.9% two, and 28.6% three (Bureau of National Statistics, 2023). These figures establish widespread multilingual competence but do not show how languages are valued or used in particular encounters. Questions of prestige, safety, and institutional access must therefore be addressed through the published studies reviewed below and, ultimately, new primary research.

Article 7 of the Constitution establishes Kazakh as the state language, provides for the official use of Russian on an equal basis with Kazakh in state organizations and local self-government, and requires conditions for the development of the languages of Kazakhstan's people (Constitution of the Republic of Kazakhstan, 1995/2024). Published scholarship interprets Kazakh as a language of statehood, Russian as a continuing interregional resource, English as mobility capital, and minority languages as unevenly institutionalized (Smagulova, 2006, 2008; Tlepbergen et al., 2025). These are differentiated forms of value, not evidence that any language has one meaning in every setting.

Minority institutional life provides useful evidence for the difference between symbolic visibility and substantive recognition. OSCE materials on multilingual and multicultural education describe schools and kindergartens in Almaty with Uyghur and Russian as languages of instruction, used as sites for maintaining mother-tongue education while also promoting knowledge of the state language (OSCE High Commissioner on National Minorities, 2018). The K. Kuzhamyarov State Republican Academic Uyghur Theater of Musical Comedy, founded in 1934, offers a long-standing cultural anchor for Uyghur-language public life (Assembly of People of Kazakhstan, 2018). The Republican State Academic Korean Theater, opened in 1932 and later relocated to Kazakhstan, performs a comparable role for the Korean community (Kazakh National Academy of Choreography, 2023). Ethnic Germans, whose demographic base declined sharply through post-1991 outmigration, retain institutional presence through the Wiedergeburt association and related cultural work aimed at preserving language, heritage, and community infrastructure (FUEN, n.d.). These examples are not simple proof of full equality. They are institutional sites where the gap between cultural display, educational access, media visibility, and participatory recognition can be examined.

Religion is included only where it intersects with ethnolinguistic boundary-making. Sharipova (2020) shows that religion and language both enter perceptions of national identity in Kazakhstan. The model therefore treats religious signs as secondary relational markers that may acquire boundary significance, not as an independent mechanism or as proof of a uniform religious hierarchy.

Official public-service information defines qandas as ethnic Kazakhs, and members of their families, who were not previously citizens and returned to their historical homeland with the relevant legal status (eGov.kz, 2026). Ministry-based reporting stated that 1,146,400 ethnic Kazakhs had returned since 1991 (Qazinform, 2024). Research on return migration describes contested belonging even among co-ethnics (Diener, 2009; Bonnenfant, 2012). The model therefore treats accent, mobility history, and familiarity with urban institutions as candidate indicators of minoritization, not as findings established by this article.

The Assembly of People of Kazakhstan is an important element in the institutionalization of interethnic harmony. Officially, it is presented as a mechanism for unity, tolerance, and intercultural dialogue. For scholarly analysis, however, it should be approached more critically. As Adams (2010) and Cummings (2012) argue for Central Asian states more broadly, state-led cultural pluralism can simultaneously provide public space to ethnocultural groups and channel them into a controlled performance of diversity. The key analytical question is whether such institutions create participatory recognition, allowing minorities to influence public norms and institutional access, or whether they primarily produce symbolic incorporation through festivals, rituals of unity, and official discourse.

A Bureau of National Statistics release reported 20,499,822 residents at the beginning of 2026, including 13,085,110 urban and 7,414,712 rural residents; it listed Kazakhs at 14,664,202 (71.5%), Russians at 2,943,022 (14.4%), and Uzbeks at 695,557 (3.4%; Bureau of National Statistics, 2026). These are current estimates rather than census results. They justify treating urban settings as important sites for future testing, but they do not show how language or ethnic boundaries are negotiated there.

Taken together, these sources define the demographic and institutional setting in which the model is applied. They do not by themselves establish whether tolerance is silent or recognitional; that distinction concerns the mechanisms and indicators specified in Section 5.

Table 3 summarizes the demographic, legal, and institutional anchors of the Kazakhstan case.

**Table 3:** Contextual anchors for the Kazakhstan case.

Indicator	Specific data or institutional feature	Analytical significance
Ethnic composition, 2021 census	Kazakhs: 70.4%; Russians: 15.5%; Uzbeks: 3.2%.	Multiethnicity coexists with the demographic majority of the titular nation.
Language regime and multilingualism	Kazakh is the state language; Russian is officially used in state organizations; 24.5% know one language, 44.9% two, and 28.6% three.	Legal balance and symbolic hierarchy shape access, status, and everyday accommodation.
Minority institutional life	Uyghur and Korean theaters, minority-language schooling, and German cultural association.	Institutional visibility is documented, but whether it constitutes substantive recognition remains an empirical question.
Qandas repatriation	Legal status for returning ethnic Kazakhs; more than 1.1 million returnees since 1991 reported in 2024.	Co-ethnics may still be minoritized through accent, mobility history, and urban codes.
Urbanization and institutional harmony	Beginning-2026 estimate: 20.5 million people, including 13.1 million urban residents; the Assembly formalizes diversity.	Cities and state-led pluralism are key arenas of everyday boundary work.

## 5 Conceptual model: four mechanisms of everyday tolerance

Synthesizing the theoretical and contextual materials reviewed above, the article proposes a conceptual model of everyday tolerance in Kazakhstan as it is represented in the scholarly literature and open sources. The model explains four mechanisms—linguistic accommodation, civic framing, institutional recognition, and normalization through interaction—that soften, reactivate, translate, invisibilize, or hierarchize boundaries in specific situations. Digital space is not treated as a separate fifth mechanism, but as a faster and more visible arena in which all four mechanisms operate. The model is a conceptual proposition for future empirical research, not itself an empirical finding.

Table 4 summarizes the four mechanisms, their indicators, and their principal risks.

**Table 4:** A conceptual model of everyday tolerance as racialized ethnic boundary work.

Mechanism	Boundary process	Concrete indicators	Condition supporting recognitional tolerance	Risk
Linguistic accommodation	Boundary softening/reactivation/ hierarchization	Language switching; reactions to accent; Kazakh/Russian/English/ minority language use; service, school, workplace, and online interactions.	Language choice is based on mutual respect, accessibility, and practical flexibility without devaluation.	One language becomes a measure of normality, loyalty, urbanity, or competence.
Civic framing	Boundary translation into civic categories	“Kazakhstani” self-identification; civic rights; common-state discourse; patriotic rituals.	The civic frame incorporates ethnic difference into equal-rights belonging.	Civic rhetoric conceals linguistic or minority inequalities.
Institutional recognition	Boundary legitimation/symbolic incorporation/public recognition	Schools, universities, media, Assembly, language rights, public service, textbooks.	Institutions provide equal participation and real accessibility, not only symbolic representation.	Diversity is reduced to festivals, folklore, or state-managed harmony.
Normalization through interaction	Boundary normalization/ invisibilization	Mixed friendships, neighborhoods, workplaces, families, universities, digital discussions, shared projects.	Interaction occurs under equal status, common goals, cooperation, and institutional support.	Contact remains polite but unequal, producing silent tolerance rather than recognition.

### 5.1 Linguistic accommodation: language choice can soften, reactivate, or racialize boundaries

The first mechanism is linguistic accommodation. In the model, choices among home, educational, administrative, and online repertoires are potentially boundary-relevant because they can index status or belonging. The mechanism does not assume that every language switch is political; it asks when switching preserves equal access and when it communicates devaluation.

Published empirical work supports this interpretation while setting limits on it. Smagulova (2008) links language attitudes to prestige, ethnic loyalty, and modernity; Fierman (2006) documents Kazakh-medium urban schooling within wider Russian-language environments; and Rees and Webb Williams (2017) and Sharipova (2020) show that language, citizenship, and national identity do not map neatly onto fixed ethnic blocs. Burkhanov and Chen (2016) more narrowly show how language-based and ethnocultural positioning can shape perceptions of external groups. Together, these studies justify treating language as a medium of boundary work without classifying every linguistic difference as racialization.

The reviewed studies do not establish a single national pattern for service encounters. In the model, a switch between Kazakh and Russian is therefore used only as an analytical diagnostic: voluntary switching that preserves access indicates accommodation, whereas switching prompted by ridicule, coercion, or status assumptions would indicate hierarchy. Whether either pattern predominates requires direct observation.

Published work treats Kazakh as central to statehood and historical redress while also documenting unequal access to high-quality language education (Smagulova, 2008; Fierman, 2006). Research on qandas describes contested incorporation among co-ethnics (Diener, 2009; Bonnenfant, 2012), but the present source base does not establish how often accent or lexical variation produces devaluation. The model therefore treats ridicule, exclusion, or status judgments attached to register as indicators to be tested, not as established national patterns.

Scholarship describes Russian as a continuing interregional and urban communicative resource whose historical institutional dominance remains politically contested (Dave, 2007; Smagulova, 2006, 2008). The model therefore treats Russian as potentially boundary-softening when it enables access and boundary-reactivating when its use becomes an unmarked standard that devalues other repertoires. Which effect prevails is an empirical question.

Tlepbergen et al. (2025) identify technology, daily use, and regional variation as important to Kazakh–English bilingualism. On that basis, the model treats English primarily as mobility capital rather than an ethnic marker. Unequal access by region, school type, and household resources is proposed as a possible classed boundary for future testing, not as a causal finding of this article.

Open institutional sources document forms of public visibility for Uyghur, Korean, German, and other ethnocultural institutions. The present source base does not establish how access to education, media, or public services is distributed across languages. The model therefore distinguishes documented institutional visibility from participatory recognition and treats the latter as a question for future empirical research.

### 5.2 Civic framing: “Kazakhstani” identity relocates ethnic difference without necessarily resolving inequality

The second mechanism is civic framing. “Kazakhstani” identity does not eliminate ethnic categories, but places them inside a shared framework of statehood, citizenship, and public responsibility. This mechanism resembles boundary expansion and boundary translation: ethnic difference remains, but it is linked to a broader civic category. In this sense, civic identity also resembles what Billig (1995) calls banal nationalism: the everyday reproduction of national belonging through ordinary categories, rituals, and public language rather than only through explicit ideology.

Studies of supraethnic identity and ethnolinguistic identification show that civic and ethnic attachments can coexist (Rees and Webb Williams, 2017; Sharipova, 2020; Zharkynbekova et al., 2025a). The model therefore asks how the formula “we are Kazakhstanis” functions in a given setting: it may widen equal belonging, or it may close discussion of linguistic or minority inequality. This is a literature-derived analytical distinction, not a claim that classrooms, workplaces, or public events display one uniform pattern.

This distinction matters because civic identity can operate in two ways. In its recognitional form, it allows a person to be simultaneously Kazakhstani and ethnically specific. It recognizes that citizenship is shared while ethnic, linguistic, and religious identities remain legitimate. In its silent form, civic identity may ask people not to mention inequality because “we are all Kazakhstanis.” In the first case, civic framing widens belonging; in the second, it makes boundaries less visible while leaving their effects intact.

Kymlicka's (1995) argument that minority rights need not contradict civic equality is important here. Applied to Kazakhstan, it means that civic unity becomes stronger when ethnocultural specificity is recognized not only through festivals but also through education, media, language access, and participation in public institutions. Fraser (2000) adds that recognition must be connected to status equality; otherwise, cultural difference can be publicly visible while social hierarchy remains unchanged.

### 5.3 Institutional recognition: schools, media, and the Assembly between participation and symbolic incorporation

The third mechanism is institutional recognition. Ethnic and linguistic difference is regulated not only through interpersonal behavior but also through schools, universities, media, state bodies, and public organizations. When institutions make the language, history, and culture of a group legitimate in public space, boundaries can soften through recognition. When institutions reduce difference to controlled representation, symbolic inequality may persist.

Published studies establish that school language regimes and student ethnolinguistic identities are shaped by both formal policy and wider linguistic environments (Fierman, 2006; Zharkynbekova et al., 2025a). The model does not infer a single pattern of classroom, peer, or digital language use. Instead, it identifies the allocation of authority, intimacy, mobility, and legitimacy across repertoires as dimensions for direct observation.

Indicators of educational recognition include responses to accent, whether students can use developing Kazakh or Russian without devaluation, the representation of minority histories, the distribution of English-learning opportunities, and the availability of real support for language requirements. Minority-medium schooling is one site where the difference between symbolic recognition and substantive participation can be tested. The model predicts recognitional tolerance when repertoires are treated as resources and boundary reactivation when one repertoire becomes the sole measure of normality.

Media is treated as another institutional site rather than as an empirically established effect. Future analysis should test whether Kazakh-, Russian-, English-, and minority-language media create segmented publics or provide translation across them. The criterion of recognitional tolerance is not the number of languages alone, but whether concerns circulate across language spaces and minority voices can shape the shared civic agenda.

The Assembly of People of Kazakhstan is a distinctive institution of managed diversity. Its strength is that it makes ethnocultural plurality visible and gives it a public vocabulary. Its limitation, as Adams (2010) and Cummings (2012) suggest for state-led cultural pluralism in Central Asia, is that recognition may remain state-centered and harmony-oriented. The conceptual question is whether the Assembly functions as participatory recognition, where groups shape public norms and institutional access, or as symbolic incorporation, where diversity is displayed within a controlled narrative of unity. This tension is not a reason to ignore the institution; it is precisely why it must be analyzed as a site of boundary management.

### 5.4 Normalization through interaction: why mixed environments do not automatically produce recognition

The fourth mechanism concerns repeated interaction across schools, universities, neighborhoods, workplaces, public services, families, and digital platforms. Contact can normalize difference, but the model does not assume that contact alone produces recognition.

Intergroup contact theory predicts prejudice reduction when interaction occurs under relatively equal status, common goals, cooperation, and institutional support (Allport, 1954; Pettigrew and Tropp, 2006). Applied as a proposition for Kazakhstan, this means that a mixed classroom, workplace, or neighborhood may still reproduce hierarchy when one group's language, accent, or cultural norm operates as the unmarked standard. Putnam (2007) further cautions that diversity may be associated with lower trust in some contexts, while Tajfel and Turner (1979) explain why status comparison can persist within peaceful interaction.

The present source base does not support a direct claim about apartment-neighborhood relations. Neighborhoods are therefore proposed as a future test site: cooperative contact under equal-status conditions should move interaction toward recognition, whereas civility accompanied by accent ridicule, linguistic coercion, or status stereotypes would indicate silent tolerance. This formulation is a hypothesis, not an observation.

Family and intimate settings are also proposed for future research. Mixed marriages, kinship relations, and interethnic friendship networks can test whether boundaries remain softened when questions of language transmission, religion, surname choice, or relations with older generations arise. The article does not claim a national pattern in these domains.

Digital platforms are likewise treated as a future empirical arena. Language choice, humor, ridicule, moderation, and counter-speech can be coded as indicators of boundary softening or hierarchy. Because no new digital corpus is analyzed here, the article makes no claim about the prevalence or direction of these processes in Kazakhstan.

## 6 Discussion: Kazakhstan in comparative perspective

Kazakhstan should not be treated as an isolated case, but it should not be compared mechanically with other multilingual societies either. Quebec, Belgium, and the Baltic states are selected not as direct analogs, but as contrast cases that clarify three different language-boundary regimes: protected titular or common language, territorial multilingualism, and post-Soviet state-language integration. These comparisons sharpen what is distinctive about Kazakhstan: its combination of state-language strengthening, Russian-language continuity, civic “Kazakhstani” belonging, minority visibility, and managed institutional pluralism. Its comparative value becomes clearer across four dimensions: the legal organization of language, the status of a titular or historically protected language, the treatment of minorities, and the relationship between civic identity and recognition.

The source-based synthesis suggests that racialized ethnic boundaries can operate without open conflict or an explicit public vocabulary of race. The proposed indicators include language hierarchy, accent evaluation, minority visibility, regional stereotypes, qandas status, and access to mobility resources (Goldberg, 2002; Hirsch, 2002; Weitz, 2002). This does not imply that Kazakhstan is structured like Western settler-colonial or post-imperial societies. It specifies a testable process through which ethnic and linguistic signs may become attached to unequal belonging where race is weak as a public category.

Quebec offers a useful comparison because French-language protection is explicitly framed as a matter of cultural survival, common public language, and historical justice. Bill 96, adopted in 2022, affirms French as Quebec's only official language and as the common language of the Quebec nation (National Assembly of Quebec, 2022). Later government guidance similarly emphasizes the modernization of the Charter of the French Language and the exemplary role of the civil administration in using and promoting French (Government of Quebec, 2025). The comparison with Kazakhstan is not exact, but it is instructive: in both cases, the strengthening of a historically vulnerable or symbolically central language can function as recognition. At the same time, it can become a status filter if access to education, employment, public services, or full belonging is tied too narrowly to one dominant linguistic standard.

Belgium offers a different comparison. Dutch, French, and German are linked to constitutionally defined language regions, with Brussels organized as bilingual; scholarship frames the arrangement through territoriality and freedom of language (van der Jeught, 2017). Kazakhstan is less territorially segmented. The model therefore expects everyday repertoire negotiation to matter more where language use is organized situationally rather than primarily by territory. This is a comparative proposition, not evidence that all Kazakhstani encounters follow one pattern.

The Baltic states provide a post-Soviet comparison in which citizenship, state-language competence, and Russian-speaking minority status became much more explicitly connected. Studies of Russian-speaking minorities in the Baltic context show how official-language requirements and citizenship regimes can become central to integration debates (Alijeva, 2017; Cheskin, 2015; May, 2012). Kazakhstan's path has been less categorial in formal citizenship terms and more reliant on the civic language of “Kazakhstani” belonging. Yet that softer civic framing does not remove the problem of recognition. It can still conceal linguistic or symbolic inequality if minority difference is acknowledged mainly through harmony rhetoric rather than equal institutional participation.

The comparison yields a source-based interpretation rather than a verdict on Kazakhstan. Multilingual stability is linked in the literature to state-language strengthening, Russian-language continuity, civic identity, minority visibility, urban interaction, and managed institutional pluralism. The model explains how these arrangements may mute, translate, or normalize racialized ethnic boundaries without naming them as race or producing open conflict.

The distinction between silent tolerance and recognitional tolerance clarifies the article's conceptual stake. These are not fixed types of society, but positions on a continuum. The same institution, classroom, workplace, family, or online community may move between them depending on power, context, and interaction. Silent tolerance can sustain everyday peace, but it may also normalize silence around inequality. Recognitional tolerance is more demanding because it requires difference to be publicly legitimate and institutionally supported.

For policy and research, the central question is not whether Kazakhstan is tolerant or intolerant. The more precise question is how stability is maintained, and where it depends on silence rather than recognition. This shifts the analysis from praise or criticism toward a sociology of how boundaries actually work.

Table 5 operationalizes this distinction as a set of indicators for future empirical testing.

**Table 5:** Indicators of silent tolerance and recognitional tolerance for future empirical testing.

Dimension	Indicators of silent tolerance	Indicators of recognitional tolerance	Possible data
Linguistic behavior	Avoiding language-related demands; silent reactions to accent; forced switching; embarrassment around imperfect speech.	Open articulation of language needs; treating accent as repertoire; multilingual accessibility.	Interviews, observation, survey of language practices.
Ethnic difference	General formulas such as “we have no ethnic problem”; treating ethnic topics as dangerous or impolite.	Discussion of ethnic difference on an equal-status basis; public visibility of groups as normal.	Focus groups, open-ended survey questions, discourse analysis.
Institutional level	Festive representation exists, but language and legal accessibility remain weak.	Concrete mechanisms of equal participation in schools, media, public service, and organizations.	Document analysis, institutional case studies, interviews.
Digital environment	Ethnic jokes and language stereotypes are dismissed as “just jokes”; little moderation or counter-speech.	Responsible multilingual dialogue; norms against devaluation; inclusive online communities.	Social media corpus, thematic and content analysis.
Mobility and class	English or urban repertoires function as hidden status filters.	Access to mobility languages is supported across class, region, and school type.	Education data, interviews, survey, regional comparison.

## 7 Limitations and directions for future research

As a Conceptual Analysis, the article relies on existing theory, published scholarship, and open contextual sources. It does not measure residents' current experiences through interviews, surveys, ethnography, or digital-corpus analysis. The limitation is therefore not hidden: the model clarifies concepts and mechanisms before primary research tests their distribution, strength, and causal relationships. The source base is also weighted toward English-language and internationally indexed scholarship; Kazakh- and Russian-language publications were not comprehensively sampled and should be addressed in a future multilingual review.

The model is deliberately abstract and cannot predict which practices dominate in Almaty, Astana, Shymkent, northern or southern regions, border towns, Russian-speaking urban networks, or minority-language communities. Future studies should test it regionally and institutionally rather than assume a single national pattern.

Future research should combine four forms of evidence: interviews or ethnography on language choice, accent, qandas status, civic identity, and minority belonging; surveys of contact, trust, perceived discrimination, civic identity, and language capital; social-media content analysis of boundary softening, ridicule, hierarchy, and polarization; and institutional case studies of schools, universities, media, and public services. Sampling should be regionally and institutionally stratified. Table 5 provides candidate indicators, while document analysis and content analysis can follow established procedures (Bowen, 2009; Krippendorff, 2019).

Three propositions correspond directly to the conceptual questions. First, multilingual accommodation should soften boundaries when it preserves mutual access and equal status; coercive switching or devaluation should reactivate them, with effects varying by region, class, and school type. Second, “Kazakhstani” civic identification should support recognitional tolerance only when paired with perceived institutional equality and legitimate expression of ethnolinguistic difference; otherwise, it may sustain silent tolerance. Third, repeated intergroup contact should support recognition under equal status, cooperation, common goals, and institutional support, whereas polite but unequal contact should reproduce hidden endurance. These propositions are not tested here.

Together with the indicators in Table 5, these propositions provide an explicit bridge from the conceptual model to future empirical designs without converting the present synthesis into an empirical study.

Such research involves ethical challenges. Ethnic and linguistic topics are sensitive, and respondents may give socially desirable answers. Researchers should use neutral wording, ensure voluntary participation, protect anonymity, and avoid questions that force respondents into rigid ethnic categories. In social media research, scholars should protect personal data, avoid reproducing harmful stereotypes, and comply with platform-specific ethical requirements.

## 8 Conclusion

The source-based analysis moves beyond treating official harmony or the absence of open conflict as sufficient evidence of tolerance. It directs attention to language choice, accent evaluation, self-naming, the discussability of difference, civic framing, and institutional access. These are proposed as mechanisms and indicators linking everyday interaction to state rhetoric, language policy, education, media, urbanization, and law.

The article interprets tolerance through racialized ethnic boundary-making. Boundaries may be softened, translated, invisibilized, or reproduced within multilingual and civic space. Kazakh, Russian, English, and minority languages carry different forms of symbolic capital; “Kazakhstani” civic identity may widen shared belonging or conceal inequality; and contact may reduce prejudice only under equal-status conditions.

The central contribution is the distinction between silent and recognitional tolerance. Silent tolerance can preserve peace while leaving unequal status unspoken; recognitional tolerance requires public legitimacy, institutional accessibility, and equal-status participation. The practical implication is that state-language strengthening should expand participation, civic identity should accommodate ethnocultural difference, and institutions should move beyond symbolic representation toward meaningful access.

Kazakhstan is therefore offered as a comparative conceptual case, not as an empirically settled model of harmony or domination. Future studies should test the indicators in Table 5 across regions, institutions, families, and digital environments to identify where stability rests on silence and where it becomes equal-status recognition. The aim is to analyze harmony as a contested social process rather than repeat it as a slogan.
